# The AirView Study: Comparison of Intubation Conditions and Ease between the Airtraq-AirView and the King Vision

**DOI:** 10.1155/2015/284142

**Published:** 2015-06-16

**Authors:** Patrick Schoettker, Jocelyn Corniche

**Affiliations:** Department of Anesthesiology, University Hospital Vaud (CHUV), Rue du Bugnon, 1011 Lausanne, Switzerland

## Abstract

We conducted a study assessing the quality and speed of intubation between the Airtraq with its new iPhone AirView app and the King Vision in a manikin. The primary endpoint was reduction of time needed for intubation. Secondary endpoints included times necessary for intubation. 30 anaesthetists randomly performed 3 intubations with each device on a difficult airway manikin. Participants had a professional experience of 12 years: 60.0% possessed the Airtraq in their hospital, 46.7% the King Vision, and 20.0% both. Median time difference [IQR] to identify glottis (1.1 [−1.3; 3.9] *P* = 0.019), for tube insertion (2.1 [−2.6; 9.4] *P* = 0.002) and lung ventilation (2.8 [−2.4; 11.5] *P* = 0.001), was shorter with the Airtraq-AirView. Median time for glottis visualization was significantly shorter with the Airtraq-AirView (5.3 [4.0; 8.4] versus 6.4 [4.6; 9.1]). Cormack Lehane before intubation was better with the King Vision (*P* = 0.03); no difference was noted during intubation, for subjective device insertion or quality of epiglottis visualisation. Assessment of tracheal tube insertion was better with the Airtraq-AirView. The Airtraq-AirView allows faster identification of the landmarks and intubation in a difficult airway manikin, while clinical relevance remains to be studied. Anaesthetists assessed the intubation better with the Airtraq-AirView.

## 1. Introduction

Numerous anatomically shaped indirect laryngoscopes are available on the market. Both the Airtraq© (Prodol Meditec SA, Vizcaya, Spain) and the King Vision (King Systems, Noblesville, IN, USA) allow better glottis visualization and Cormack Lehane score (CL) than direct laryngoscopy [1], a fast learning curve [2], and they both offer a blade that incorporates a tube channel that holds the endotracheal tube (ETT) and guides it towards the glottis [3]. Visualization of the anatomical landmarks and ETT movement and passage is obtained either by direct vision on the device [4] or via a LCD display [5, 6]. The high-quality enlarged images obtained through these different monitors have been shown to be useful tools for training and teaching [7–9] and for managing difficult airway [3], allowing for better coordination between operator and assistant. Recently, a specially designed clip-on wireless camera that relays the image on a separate monitor screen has been commercialized for the Airtraq, improving the ease of tracheal intubation [10].

Smartphones have become increasingly popular among anaesthetists [11] and are carried in pockets and thus readily available. They possess high-quality camera optics allowing image viewing and acquisition, storage, manipulation, and transmission leading to regular usage in association with clinical applications in theatres [12, 13]. A recent study has assessed the usefulness of the iPhone (Apple Inc., Cupertino, CA, USA) as an adjunct aid to assist in fibreoptic intubation and clinical teaching in a difficult airway scenario when a screen for video-assisted bronchoscopy was unavailable [14]. Although it was found more difficult to use compared to a bronchoscope, the iPhone modified bronchoscope offered several advantages for teaching fibreoptic technique.

**Figure 1:**
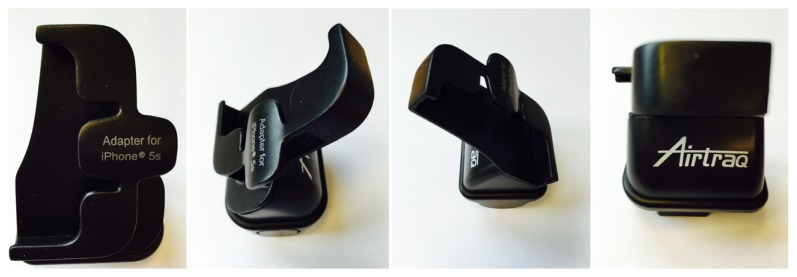


Recently, an iPhone app (AirView by Mobilemed Sàrl, Switzerland) that allows live visualisation of the intubation process with the Airtraq has been made freely available on the app store. It works in conjunction with a specially designed adapter (A-308) for iPhone 5s, designed and manufactured by Prodol Meditec Limited, Coast, Zhuhai, Guangdong, China, and distributed through the Airtraq worldwide distributors network (Prodol Meditec SA, Las Arenas, Spain) (see Figure 1).

The aim of this study was to compare success rate, time to ventilation, and quality of intubation between the Airtraq coupled to an iPhone using the new AirView app and the King Vision in a manikin study simulating a difficult airway. The primary endpoint was reduction in time necessary for successful intubation on the first attempt for each device. Secondary endpoints included time necessary to identify the glottis and to insert the tube and inflate its cuff, best view during laryngoscopy before and during intubation, as well as ease of insertion of the device in the mouth, of epiglottis visualisation, and of intubation.

## 2. Method

**Figure 2:**
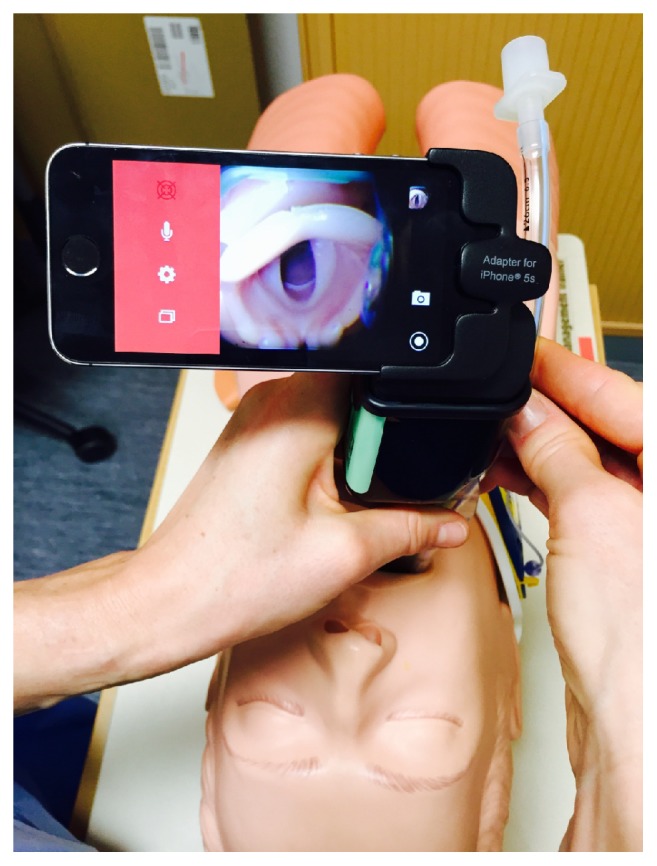


The President of the Local Institutional Ethics Committee (Professor P. Francioli, Commission Cantonale d'Ethique de la recherche sur l'être humain, 1012 Lausanne, Switzerland) judged that, according to the national guidelines for clinical research, ethics committee approval was not required. 30 senior anaesthetists attending a difficult airway course in Switzerland gave written consent to participate in this study. Each anaesthetist was given a standardized demonstration by the commercial representative of the Airtraq and King Vision device. The AirView app is downloadable free of charge on the iTunes app store and a specifically designed adaptor warrants a physical connection between an iPhone 5s and the Airtraq (see Figure 2).

All participants used the Airtraq sp (AT) size 3 coupled to an iPhone 5s and the King Vision with a size 3 channeled blade (KVC) with a preloaded lubricated 7.5 mm tube (Mallinckrodt Hi-Contour Oral Tracheal Tube Cuffed; Covidien Ilc, 15 Hampshire Street, Mansfield, MA, USA). They were assigned by envelope randomization to perform 6 intubations (3 with the Airtraq-AirView and 3 with the King Vision) on an airway manikin (ALS SkillTrainer; Laerdal, Stavanger, Norway) simulating a difficult airway with its neck immobilized by a cervical collar (Philadelphia Cervical Collar Co., Thorofare, NJ, USA), causing also reduction in mouth opening to 3 cm.

The timer was started (T_0_) when touching the AT or KVC, which was then inserted into the mouth. Once the laryngeal inlet was identified, the time was recorded (T_1_). The endotracheal tube (ETT) was advanced into the trachea, the cuff inflated (T_2_), and the timer was stopped (T_3_) when ventilation of the lungs was visible after connecting the tube to a bag-mask resuscitator (Ambu SPUR II resuscitator, Ambu A/S, 2750 Ballerup).

Insertion, intubation, and ventilation success within 60 seconds, time necessary to identify the glottis with the Airtraq (TA_1_) or the King Vision (TK_1_), to insert the tube and inflate its cuff (TA_2_) or (TK_2_), and to ventilate the lungs (TA_3_) or (TK_3_), were recorded. Time differences were defined as TK-TA and if >0, time necessary to fulfill the task using the Airtraq-AirView was shorter; if = 0, no differences in times were observed between the King Vision and the Airtraq-AirView; and if TK-TA was <0, the King Vision allowed quicker times.

Best view during indirect laryngoscopy was assessed as Cormack Lehane [15] (CL 1, 2a, 2b, 3, 4) and percentage of glottic opening (POGO) [16] before and during intubation. Ease of insertion of the device in the mouth, of epiglottis visualisation, and of intubation was also documented (1 = very easy, 2 = easy, 3 = moderate, 4 = difficult, and 5 = very difficult).

The primary endpoint was time reduction necessary for successful intubation on the first attempt for each device. A failed intubation was defined as oesophageal intubation or a time necessary to ventilate the lungs longer than 60 seconds. Secondary endpoints included time necessary to identify the glottis and to insert the tube and inflate its cuff, best view during laryngoscopy before and during intubation, as well as ease of insertion of the device in the mouth, of epiglottis visualisation, and of intubation.

Based on the study by Wetsch et al. [17], a time to ventilate of 33 seconds was deemed clinically relevant. We considered that a time difference of ten seconds (the one-third of 33 seconds) between the two devices in the primary outcome of “time to first ventilation” would be clinically relevant. To detect this difference, with a power of 90% at a two-tailed significance level of 5% (Wilcoxon-Mann-Whitney two groups test), the G ^*^ power statistical power analyses software [18] calculated a total sample size of 180 intubations.

Statistical tests used were median or chi-squared where appropriate. Data were analyzed using the JMP 10 statistical package (SAS Institute Inc, Cary, NC, USA).

## 3. Results

All anaesthetists (16 females and 14 males) had experience in indirect laryngoscopy with an average exposure to airway management of 12 years (SD 9.9).

18 (60.0%) participants used the Airtraq regularly in their clinical setting, 14 (46.7%) the King Vision, and 6 (20.0%) had access to both.

Insertion, intubation, and ventilation were possible within the allocated time in all cases with both devices by all participants.

Time necessary to identify glottis, to insert the tube and inflate its cuff, and to ventilate the lungs was significantly shorter with the Airtraq-AirView (see Table 1).

Cormack Lehane best view during videolaryngoscopy before intubation was reported as significantly better with the King Vision (*P* = 0.03) while no significant difference was noted during the intubation process (see Table 2).

A CL 1 was present in 72 (80.0%) intubations with the Airtraq-AirView versus 80 (88.9%) with the King Vision and a CL 2 was present in 18 (20.0%) AT intubations versus 10 (11.1%) for the KVC group. Median POGO was described as equal between the Airtraq-AirView group versus King Vision before (86.5% [77.3; 92.6] versus 85.8% [75.2; 91.9]) and during intubation (85.4% [76.2; 91.4] versus 84.8% [75.2; 90.8]).

No statistically significant difference was noted for subjective ease of device insertion or quality of epiglottis visualisation while subjective assessment of ease of tracheal tube insertion was significantly better with the Airtraq-AirView (*P* = 0.001) (see Table 3).

## 4. Discussion

We showed that the Airtraq coupled to an iPhone with its dedicated AirView app allowed quicker identification of epiglottis and intubation as well as easier tracheal tube insertion on an airway manikin simulating difficult intubation than the King Vision by experienced anaesthetists.

Channeled indirect laryngoscopes are popular devices in cases of difficult intubation and have been shown to reduce intubation time, improve the intubation difficulty scale, and significantly improve the view during laryngoscopy [19–22]. Improved lighting and a view through a screen has been shown to facilitate tracheal inlet visualization [23, 24]. Only individual solutions have been reported on adding variable types of cameras and monitors to these devices [25–27], while using an existing optional video camera for the Airtraq has been shown to improve the ease of tracheal intubation in specific circumstances [10, 28]. We provide the first report about a visualization and intubation app accessible with no charge through the app store and tailored for an existing indirect laryngoscope, allowing direct view with the help of a specific adaptor and comparing its use with a high-resolution videolaryngoscope.

Although all intubations were possible within the allocated time with both devices, times necessary to identify mandatory anatomical landmarks for proper intubation were significantly shorter as was the subjective assessment of tracheal tube insertion with the Airtraq coupled to the AirView app. Ueshima and Asai [29] report even shorter intubation times while using the Airtraq. Their study was designed to compare the ease of tracheal intubation with various external lighting conditions with a regular airway manikin. In another study assessing success rates and endotracheal tube insertion times in a difficult airway setting, Wetsch et al. [30] found that 43 seconds was necessary for intubation with the Airtraq. Although it also used experienced anesthesiologists, their study setting varied as their manikin was trapped in a car, simulating difficult airway and difficulty in airway access. Our study was designed to simulate a difficult airway scenario with a manikin positioned supine on a table, as happening in an hospital setting, similarly to a second study by Wetsch et al. [17], showing same results as ours.

The Cormack Lehane view obtained during the indirect laryngoscopy was reported better with the King Vision while no difference was noted during intubation. Yun et al. [31] compared Cormack Lehane views between different videolaryngoscopes in a tactical setting and were unable to show a difference between the Airtraq and the King Vision. Their setting included paramedics and using the Airtraq with its eyepiece, therefore not allowing external viewing of the intubation process. The percentage of glottic opening is the favored classification in assessing visualization during videolaryngoscopy. It essentially provides a continuous and numerical value across the full range of Cormack Lehane grades I through III, making it more reliable in assessing laryngeal exposition. Assessing performance of indirect laryngoscopes includes vocal cords visualization but focuses more on successful intubation, time necessary for intubation, and the complexity of the maneuver [32]. In our study, shorter time of ventilation and easier intubation with the Airtraq coupled to AirView show promising results.

There are several limitations in our study. First of all, difficult airway research on manikins relies mainly on how realistic the upper airway of the manikin is. New airway devices must be assessed in an objective way without patient harm and manikin-based airway research is accepted [4, 33–35]. Studies have reported considerable disparity in airway anatomy between manikins and actual patients [34]. Because the upper airway anatomy of the manikin used in this study has not been evaluated, the results obtained may be valid only in the manikin we used. Second, blinding of each participant to the device used for intubation is impossible. Some participants may have a preference for one of the two devices studied before the study. In our setting, slightly more anesthesiologists had experience with the Airtraq, while none had had a chance to practice intubation with the AirView app. Third, we conducted this study during a difficult airway course, where mainly experienced anesthesiologists were present with an interest in difficulty airway management. The results obtained may not be reproduced with more junior providers or paramedics. And fourth, the clinical relevance of the time difference found in our study may be irrelevant, even if similar intubation times have been described in different manikin settings [36].

Finally, smartphones are carried by most physicians throughout hospitals and in the operating rooms [11], in both developed and developing countries [37]. The addition of an iPhone to an Airtraq provides a high-quality vision, allowing image manipulation and analysis, recording, and postoperative sharing for teaching purpose, while not modifying the line of sight. However, further thoughts must be given to legal issues, such as recording patient data on a smartphone, private or not. Some countries have enforced laws, such as the Health Information Privacy and Accountability Act (HIPPA) (http://www.hhs.gov/ocr/privacy) governing the use, storage, and dissemination of personal health information. It therefore protects the privacy of an individual's health information and governs the way certain health care providers and benefits plans collect, maintain, use, and disclose protected health information.

We conclude that the Airtraq-AirView allows faster identification of the landmarks and intubation in a simulated difficult airway manikin in comparison to an existing high-quality videolaryngoscope. Anaesthetists assessed the intubation to be better with the Airtraq-AirView.

Clinical trials evaluating the effects of a specially designed app associated to the Airtraq on intubation success in the clinical setting are underway.

## Figures and Tables

**Table 1:** 

	Airtraq-AirView *n* = 90		King Vision *n* = 90		Delta K-A		*P*
	Median	IQR	Median	IQR	Median	IQR	
Time to identify glottis	5.3	[3.9; 8.4]	6.4	[4.6; 9.1]	1.1	[−1.3; 3.9]	0.019
Time to inflate the cuff	14.2	[9.1; 18.2]	15.2	[10.9; 26.1]	2.1	[−2.6; 9.4]	0.002
Time to ventilate the lungs	16.6	[11.9; 21.1]	17.9	[13.6; 28.5]	2.8	[−2.4; 11.5]	0.0002

Times are expressed in seconds.

**Table 2:** 

Cormack Lehane best view	Airtraq-AirView		King Vision	
	*n* = 90	%	*n* = 90	%
1	70	77.8	81	90.0
2a	20	22.2	8	8.9
2b	0		1	1.1

**Table 3:** 

	Ease of device insertion		Ease of epiglottis visualisation		Ease of intubation	
	AT-AirView *n* = 90	King Vision *n* = 90	AT-AirView *n* = 90	King Vision *n* = 90	AT-AirView *n* = 90	King Vision *n* = 90
1	51	48	66	68	63	52
2	33	34	24	20	22	15
3	6	8	0	1	5	14
4	0	0	0	1	0	9
